# Viral metagenomic characterization of HAdV-B-related signals in bronchoalveolar lavage fluid from pediatric and adult clinical cohorts

**DOI:** 10.3389/fpubh.2026.1961482

**Published:** 2026-10-01

**Authors:** Chunyan Zhao, Jiaheng Chen, Ping Wu, Wen Zhang, Songyi Ning

**Affiliations:** 1 School of Health and Nursing, Wuxi Taihu University, Wuxi, China; 2 School of Medicine, Jiangsu University, Zhenjiang, China; 3 Department of General Surgery, Wujin Hospital Affiliated with Jiangsu University, Changzhou, Jiangsu Province, China

**Keywords:** bronchoalveolar lavage fluid, HAdV-B, human adenovirus, metagenomic next-generation sequencing, pediatric pneumonia

## Abstract

Human adenovirus (HAdV) is an important respiratory virus in children, but sequence-level evidence for HAdV-related signals in lower respiratory tract samples remains limited. In this study, we analyzed metagenomic next-generation sequencing data from 189 bronchoalveolar lavage fluid (BALF) libraries from pediatric and adult clinical cohorts, including 30 pediatric and 159 adult patients, together with laboratory environmental controls. MEGAN read-level classification was used to screen for *Adenoviridae*-related signals. HAdV-related contigs were further reviewed, and a BALF-derived HAdV-B-related representative contig was selected for read recruitment and coverage analysis. Phylogenetic trees were reconstructed using penton base, hexon, and fiber protein sequences. *Adenoviridae*-classified signals were more apparent in pediatric BALF libraries than in adult BALF libraries or environmental controls. Several pediatric BALF libraries showed broad read coverage across the HAdV-B-related representative contig, whereas adult libraries and environmental controls mainly showed low-coverage-breadth signals. Phylogenetic analysis placed BALF-derived penton base, hexon, and fiber sequences within HAdV-B-related lineages. Twenty-seven capsid protein sequences from 12 pediatric samples had best BLASTp matches to references annotated as HAdV-B3 (*n* = 23) or HAdV-B7 (*n* = 4). These findings provide sequence evidence for HAdV-B-related sequence signals in clinically selected pediatric BALF samples and support further investigation using targeted assays and genome sequencing.

## 1 Introduction

Pneumonia is a common and clinically important lower respiratory tract infection in both children and adults. In practice, the challenge is often not simply to determine whether infection is present, but to interpret pathogen signals accurately in complex clinical samples. The pathogen spectrum of pediatric pneumonia varies with age, season, geographic region, and diagnostic approach, and viruses represent an important component of community-acquired pneumonia requiring hospitalization in children (1). The etiological composition of adult pneumonia is also complex, and clinical interpretation usually requires consideration of sample type, disease severity, and microbiological findings (2). Culture, antigen testing, PCR, and multiplex PCR panels remain important tools in routine pneumonia diagnostics, but these methods usually rely on predefined targets and may not cover all potential pathogens. For pneumonia samples with negative routine tests, discordant results, or complex microbial signals, untargeted sequencing can provide additional information for pathogen profiling.

Etiological studies of pediatric pneumonia have shown that viruses are a key pathogen group in lower respiratory tract infections. A study of hospitalized children with community-acquired pneumonia in the United States reported frequent viral detection and age-related differences in pathogen distribution (3). The PERCH multi-country case-control study also showed that the causes of severe childhood pneumonia are not attributable to a single pathogen group, as viruses, bacteria, and mixed infections may all contribute to disease (4). These findings suggest that pathogen analysis in pediatric pneumonia should not rely solely on conventional bacterial culture, but should also include molecular assays and broader pathogen screening strategies. For virus-associated pneumonia, sample source, detection strategy, and downstream sequence interpretation can all affect the reliability of the results.

Human adenoviruses (HAdVs) belong to the genus *Mastadenovirus* and are non-enveloped, icosahedral viruses with linear double-stranded DNA genomes that replicate in the host-cell nucleus (5, 6). Respiratory disease is commonly associated with HAdV-B, HAdV-C, and HAdV-E, whereas HAdV-D types such as 8 and 37 are associated with epidemic keratoconjunctivitis and HAdV-F40 and HAdV-F41 predominantly cause gastroenteritis (6). Respiratory manifestations include fever, cough, and sore throat and range from upper respiratory tract illness to pneumonia; severe or disseminated infection is of particular concern in immunocompromised patients (5, 6). In murine cells exposed to recombinant HAdV-5 vectors, cGAS-STING-TBK1 signaling contributes to IRF3 activation and type I interferon responses (7). These findings provide biological context for HAdV sequence characterization; host pathway activity was not assessed in the present study.

HAdV-B-related types have attracted considerable attention in studies of pediatric respiratory infections. Studies of acute respiratory infections in Chinese children have shown that HAdV-B3, HAdV-B7, and some HAdV-C-related types are commonly detected in pediatric respiratory samples (8). A study of pediatric acute respiratory tract infections in Guangzhou from 2010 to 2021 also showed temporal variation in the distribution of HAdV types (9). In pediatric acute respiratory illness cohorts, the composition and clinical features of different HAdV species are not completely consistent (10). Some studies have also suggested that HAdV-7-associated pneumonia may be linked to a more severe clinical course (11). These findings indicate that HAdV-B-related signals in pediatric pneumonia samples deserve further sequence-level characterization.

HAdV-related signals are not restricted to pediatric respiratory infections. A study of adults with acute respiratory infection in China detected HAdV-B, HAdV-C, and HAdV-E, suggesting that HAdV-related signals can also be present in adult respiratory samples (12). Including both pediatric and adult BALF cohorts within the same study framework may therefore help describe how HAdV-related signals differ across age-related and clinical sample backgrounds. However, pediatric and adult clinical cohorts may differ in immune status, underlying diseases, pathogen spectrum, and clinical indications for sampling. For this reason, such findings are better interpreted at the level of sample groups and sequencing signals, rather than being simplified into a direct comparison of clinical prevalence.

Bronchoalveolar lavage fluid (BALF) provides an important sample source for analyzing viral signals in the lower respiratory tract. Compared with nasopharyngeal or throat swabs, BALF is closer to the pulmonary lesion site and can more directly reflect microbial and viral nucleic acid signals in the lower respiratory tract. Studies of pediatric lower respiratory tract infections have shown that BALF metagenomic next-generation sequencing (mNGS) can identify multiple categories of pathogens in complex pulmonary infection samples and can complement conventional diagnostic methods (13). Another study also noted that BALF has long been regarded as an important sample type for detecting lower respiratory tract infections, although its collection is usually limited by clinical indications (14). Virome analysis using residual clinical BALF samples can therefore provide a sequence-level window into lower respiratory tract viral signals without requiring additional invasive sampling.

mNGS offers a more open strategy for pathogen profiling in pneumonia samples. Because mNGS does not depend on predefined targets, it can capture nucleic acid signals from bacteria, fungi, viruses, and other microorganisms, making it suitable for pathogen exploration in complex infection samples (7). For virome studies, the value of mNGS lies not only in read-level classification, but also in the ability to combine *de novo* assembly, read recruitment, and phylogenetic analysis for deeper sequence-level interpretation of candidate viral signals. Molecular typing and phylogenetic placement of HAdV also require sufficient sequence evidence. Traditional HAdV classification once relied on serological methods, but modern HAdV naming and classification increasingly emphasize genome sequences and phylogenetic information (16). Hexon, penton base, and fiber are commonly used major capsid genes for HAdV typing and phylogenetic analysis, as these regions are closely related to antigenicity, cell binding, and viral entry. For HAdV-related fragments assembled from mNGS data, when complete genomes cannot be obtained for all samples, phylogenetic analysis based on multiple major capsid genes can provide a more cautious basis for lineage placement.

Several studies have described the molecular epidemiology of pediatric respiratory HAdV across different regions and clinical sample types. Many of these studies focused mainly on nasopharyngeal swabs, throat swabs, or other upper respiratory tract samples, and their core analyses often involved typing and phylogenetic analysis of PCR-positive samples (17, 18). Other studies have explored the utility of BALF mNGS for pathogen detection in pediatric pneumonia or lower respiratory tract infections, suggesting that BALF mNGS can serve as a complementary approach to routine pathogen testing (19, 20). However, virome-level descriptions of HAdV-related signals in BALF from pneumonia patients remain relatively limited. In particular, few studies have integrated read-level classification, assembly-supported contigs, representative contig read recruitment, and phylogenetic analysis of major capsid genes using BALF mNGS data. This gap limits our understanding of the sequence characteristics and phylogenetic background of HAdV-related signals in lower respiratory tract samples.

Based on this background, the present study included BALF mNGS libraries from pediatric and adult clinical cohorts, together with laboratory environmental controls, to perform an initial screen for *Adenoviridae* read-level classification signals. We then further screened HAdV-related contigs, selected a BALF-derived HAdV-B-related representative contig for read recruitment and coverage breadth analysis, and performed phylogenetic analysis based on the three major capsid proteins, penton base, hexon, and fiber. This study aimed to describe the metagenomic features of HAdV-B-related signals in BALF at multiple levels, including read classification, assembly, read recruitment, and phylogenetic placement, and to provide genomic information for future studies of lower respiratory tract HAdV in clinically selected pediatric samples.

## 2 Methods

### 2.1 Sample collection and processing

From July 2025 to January 2026, a total of 189 residual BALF samples obtained during routine clinical care were included in this observational virome analysis. BALF was obtained during clinical evaluation for suspected pneumonia, although the diagnoses subsequently documented in the available records were heterogeneous. The pediatric cohort comprised 30 BALF samples from the Affiliated Children’s Hospital of Jiangnan University, whereas the adult cohort comprised 159 BALF samples, including 149 from Yixing People’s Hospital and 10 from the Affiliated Hospital of Jiangsu University (Jiangbin Hospital). The pediatric cohort included 29 patients with a recorded pneumonia-related diagnosis and one with acute bronchitis. *Mycoplasma pneumoniae*-related diagnoses were recorded in 25 of the 30 pediatric patients (83.3%). The adult cohort included 113 patients with pneumonia-related diagnoses and 46 with other recorded clinical conditions. The pediatric and adult cohorts were not designed as clinically matched comparison groups. Detailed sample-level clinical information, cohort summaries, and adult diagnostic subgroup summaries are provided in Supplementary Table S1 (Sheets S1A–S1C).

All BALF samples were collected in sterile tubes and transported on dry ice to the biosafety level 2 laboratory at the School of Medicine, Jiangsu University, for subsequent virome analysis. Three laboratory environmental surface controls were included as background references: a PCR instrument surface (Con-P-1), a centrifuge inner surface (Con-L-1), and a biosafety cabinet inner surface (Con-S-1). The three laboratory environmental surface controls were collected using sterile swabs and subsequently subjected to nucleic acid extraction, library preparation, and sequencing using the corresponding virome workflow. For sample processing, frozen BALF samples were thawed on ice. A 600 μL aliquot of each original sample was transferred to a new sterile tube and vortexed for 5 min to ensure sufficient homogenization. The processed samples were then centrifuged at 15,000 × g for 10 min at 4 °C to remove cell debris and large particles. The supernatant was collected and stored at −80 °C before viral particle enrichment, filtration, and nucleic acid extraction.

### 2.2 Library preparation and sequencing

To enrich viral particles and reduce the influence of host-derived nucleic acids, 500 μL of preprocessed BALF supernatant was centrifuged at 12,000 × g for 5 min at 4 °C. The supernatant was then collected and passed through a 0.45 μm pore-size filter to remove residual eukaryotic cells and larger particles. For nuclease treatment, 166.5 μL of filtrate was mixed with 20 μL of 10 × TURBO DNase buffer, 7 μL of TURBO DNase (Invitrogen/Thermo Fisher Scientific, Cat. No. AM2238, 2 U/μL; 14 U per reaction), 3 μL of Baseline-ZERO DNase (LGC Biosearch Technologies), 3 μL of Benzonase nuclease (Merck/EMD Millipore, Cat. No. 70664), and 0.5 μL of RNase A, DNase- and protease-free (Thermo Scientific, Cat. No. EN0531, 10 mg/mL). The final reaction volume was 200 μL. The mixture was incubated at 37 °C for 30 min to degrade free nucleic acids that were not protected within viral particles.

After nuclease treatment, viral DNA and RNA were extracted using the QIAamp MinElute Virus Spin Kit (QIAGEN, Hilden, Germany; Ref. 57704) according to the manufacturer’s instructions. Reverse transcription was included to recover RNA-derived sequences alongside DNA-virus sequences in the same virome workflow. First-strand cDNA was synthesized from the extracted nucleic acids using SuperScript III Reverse Transcriptase (Life Technologies/Invitrogen, Carlsbad, CA, USA; Cat. No. 18080-085) and random hexamer primers. The second strand was synthesized using DNA Polymerase I Klenow Fragment (Vazyme Biotech, Nanjing, China; Cat. No. N104-01) to generate double-stranded DNA. Sequencing libraries were then prepared from the resulting double-stranded DNA using the TruePrep DNA Library Prep Kit V2 for Illumina (Vazyme Biotech, Nanjing, China; Cat. No. TD503). A total of 189 BALF sequencing libraries were constructed and sequenced on the Illumina NovaSeq 6,000 platform. Sequencing was performed using a 150 bp paired-end strategy with dual-index barcodes for sample demultiplexing.

### 2.3 Bioinformatic preprocessing, assembly, and viral sequence annotation

Raw sequencing data were first demultiplexed and paired by the sequencing platform software. Subsequent bioinformatic analyses were performed on a Linux server cluster. Raw reads were quality controlled using Trim Galore v0.6.11 (21) to remove adapter sequences, low-quality bases, and short reads (--phred33 --length 50 --stringency 3 --paired), generating quality-controlled paired-end reads. The quality-controlled reads were mapped to the human reference genome (GCF_000001405.40) using Bowtie2 v2.5.0 (22), and reads mapped to the host genome were removed to obtain host-depleted clean reads.

The host-depleted clean reads were subjected to *de novo* assembly using MEGAHIT v1.2.9 (23) (--min-contig-len 200). The assembled contigs were compared against a local viral protein database using the BLASTx mode of DIAMOND v2.1.18 (24) (-e 1e-5 -k 25 -f 100). The local viral protein database was built from NCBI viral protein sequences and updated in September 2025. The DAA files generated by DIAMOND were then imported into MEGAN v7.1.1 (25) for taxonomic annotation based on the lowest common ancestor (LCA) algorithm.

In addition to contig-level analysis, read-level viral classification was also performed. Host-depleted clean reads were compared against the viral protein subset of the NCBI NR database using DIAMOND BLASTx (taxonomy ID 10239; -e 1e-5 -k 25). To reduce false-positive classification caused by non-viral sequences, candidate viral reads from the initial screen were further checked against the complete NR protein database. The retained read-level DIAMOND results were imported into MEGAN and converted into RMA format for subsequent comparative analysis.

All 192 libraries were compared in MEGAN v7.1.1 with “Use normalized counts” and “Keep at least 1 read” enabled. Family-level Adenoviridae counts were exported and analyzed in R without further library-size normalization.

### 2.4 Screening of HAdV-related contigs and annotation of the representative contig

Based on the MEGAN classification results, contigs annotated as *Adenoviridae* were further screened. Candidate contigs were manually reviewed using Geneious Prime v2024.0.5. For HAdV-related contigs longer than 500 bp, we focused on whether they contained major capsid genes such as penton base, hexon, and fiber.

A near-complete BALF-derived HAdV-B-related representative contig was selected as the reference sequence for subsequent read recruitment analysis. This contig was named JN-BALF-025-35976 and had a length of 35,414 bp. The representative contig contained major capsid genes, including penton base, hexon, and fiber, and its genome organization was annotated in Geneious Prime. Major capsid genes were highlighted in the schematic, while other predicted open reading frames were shown as background ORFs.

### 2.5 Representative contig read recruitment and coverage analysis

To assess read-level support for the assembled HAdV-B-related representative contig, processed paired-end reads from pediatric BALF libraries, adult BALF libraries, and laboratory environmental control libraries were recruited to the representative contig. Host-depleted clean reads were used for clinical BALF samples, while quality-controlled paired-end reads were used for environmental controls. A Bowtie2 index was built from the single representative contig.

Read recruitment was performed using Bowtie2 (--very-sensitive-local --no-mixed --no-discordant --no-unal). The alignment results were filtered, sorted, and summarized using Samtools v1.13 (26). Only proper-pair alignment records were retained for downstream analysis (-f 2 -F 3844). Coverage depth was calculated using samtools depth -aa -d 0. Properly paired read pairs were used as the basic counting unit in this study. Two retained proper-pair alignment records were counted as one mapped read pair. The denominator includes all read pairs supplied to recruitment, including both recruited and unrecruited pairs. For each sample, representative-contig RPM was calculated as mapped pairs/input read pairs × 1,000,000 and was used to describe read recruitment abundance. Coverage breadth was calculated as the percentage of bases in the representative contig covered by at least one read and was used to describe the distribution of recruited reads across the contig (Supplementary Table S2).

### 2.6 Extraction of major capsid genes and phylogenetic analysis

To determine the phylogenetic placement of BALF-derived HAdV-related sequences, phylogenetic analyses were performed for three major capsid proteins: penton base, hexon, and fiber. BALF-derived sequences were extracted from HAdV-related contigs, and their target protein regions were confirmed using CDD search (27) and NCBI BLASTp results. Reference sequences were downloaded from the NCBI database and included representative human adenovirus species and major HAdV-B-related types.

Multiple sequence alignments were performed using MAFFT v7.526 (28) (--auto) and then trimmed using trimAl v1.5.0 (29) (-automated1). Maximum-likelihood phylogenetic trees were reconstructed using IQ-TREE v2.3.6 (30). The best-fit substitution model was selected automatically by ModelFinder (-m MFP), and major branch support was assessed with 1,000 ultrafast bootstrap replicates (-bb 1,000). The phylogenetic trees were finally visualized using ChiPlot (31) and Adobe Illustrator 2025 v29.0.

### 2.7 Statistical analysis and visualization

All statistical summaries and graphical visualizations were performed in R v4.3.1 (32). Data processing was mainly conducted using the dplyr, tidyr, readr, and stringr packages. Figures were generated using ggplot2, with scales and patchwork used to optimize axes, legends, and multi-panel layouts. For the descriptive comparison of MEGAN-normalized *Adenoviridae* abundance between the pediatric and adult BALF groups, a two-sided Wilcoxon rank-sum test was used. Laboratory environmental controls were displayed only as background references and were not included in this statistical comparison. A two-sided *p* value < 0.05 was considered statistically significant.

## 3 Results

### 3.1 MEGAN read classification indicated more prominent *Adenoviridae* signals in pediatric BALF libraries

The pediatric cohort included 29 patients with a recorded pneumonia-related diagnosis and one with acute bronchitis. *Mycoplasma pneumoniae*-related diagnoses were recorded in 25 of the 30 pediatric patients (83.3%). The adult cohort included 113 patients with pneumonia-related diagnoses and 46 with other recorded clinical conditions (Supplementary Table S1B). Both cohorts comprised residual clinical BALF samples processed using the same virome workflow, but they differed in sampling center and clinical composition.

To obtain an initial read-level assessment of *Adenoviridae*-related signals in BALF libraries, we compared MEGAN-normalized abundance among pediatric BALF libraries, adult BALF libraries, and laboratory environmental control libraries. *Adenoviridae*-classified abundance was more prominent in several pediatric BALF libraries, whereas signals in most adult BALF libraries and laboratory environmental controls were low or close to the background range (Figure 1A).

**Figure 1:**
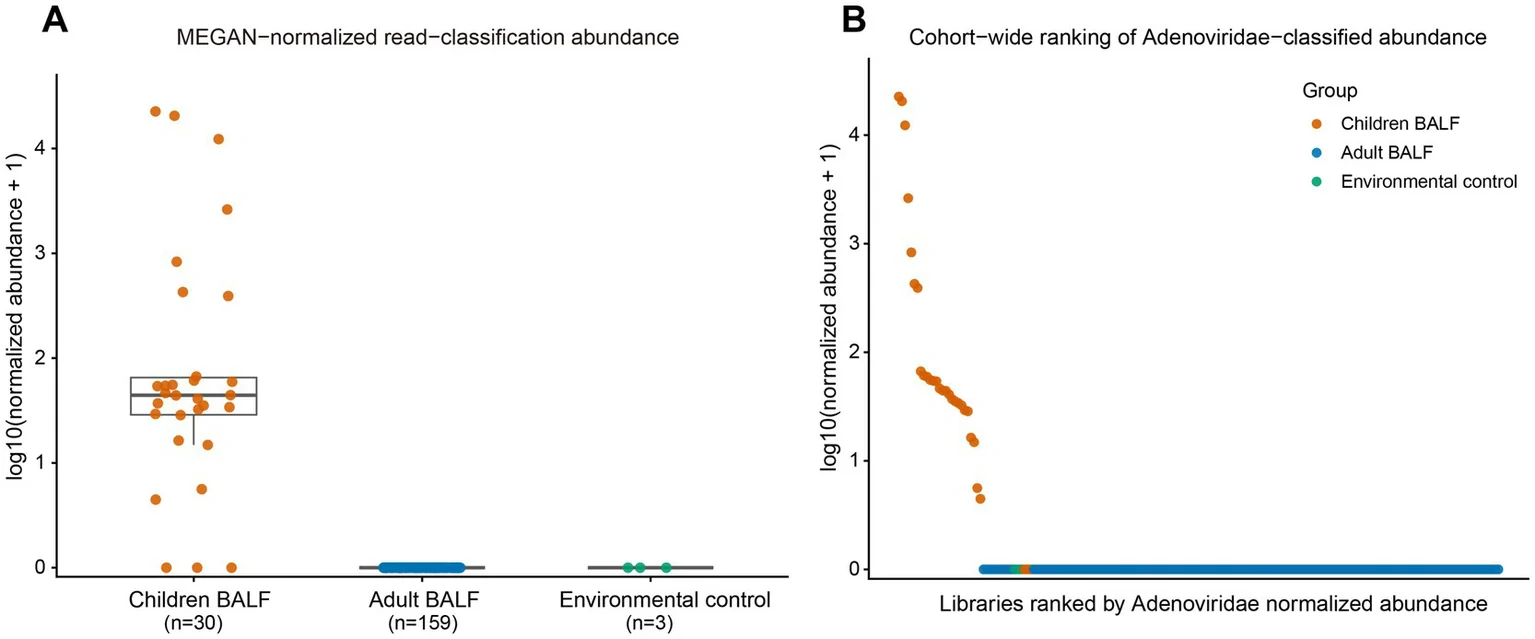
Read-level *Adenoviridae*-classified signals in BALF metagenomic libraries. **(A)** Group-level distribution of MEGAN-normalized *Adenoviridae* abundance in pediatric BALF libraries, adult BALF libraries, and laboratory environmental control libraries. Each point represents one library. The *y*-axis shows log10(normalized abundance + 1). The comparison between the pediatric and adult BALF groups was performed using the Wilcoxon rank-sum test. Environmental controls were shown only as laboratory background controls and were not included in this statistical comparison. **(B)** Cohort-wide distribution of *Adenoviridae* normalized abundance after all libraries were ranked from high to low abundance. Each point represents one library, and colors indicate sample groups. MEGAN-based read classification was used to display read-level classification signals and was not used alone for clinical HAdV infection diagnosis or specific type assignment.

We then ranked all libraries according to their *Adenoviridae* normalized abundance. Libraries with the highest *Adenoviridae* abundance were predominantly from the pediatric BALF group, whereas most adult BALF libraries and environmental controls were located in the low-abundance range (Figure 1B). The distribution of *Adenoviridae* normalized abundance differed between the pediatric and adult BALF groups (two-sided Wilcoxon rank-sum test, *p* < 0.001). Laboratory environmental controls were displayed only as background references and were not included in this statistical comparison. Because the pediatric and adult cohorts differed in clinical composition and sampling center and were not designed as clinically matched groups, this comparison was interpreted descriptively rather than as evidence of an independent age effect or age-specific HAdV prevalence. In addition, MEGAN-based classification represents screening-level taxonomic evidence from mNGS data and was therefore not interpreted alone as evidence of clinical HAdV infection or specific HAdV type assignment.

### 3.2 Representative-contig read recruitment showed broader HAdV-B-related read support in pediatric BALF libraries

Based on the read classification results, we further used assembled BALF-derived HAdV-related sequences for read-level validation. A near-complete HAdV-B-related representative contig, JN-BALF-025-35976, was selected as the reference sequence. Processed paired-end reads from all clinical and environmental-control libraries were recruited to this representative contig. The representative contig was 35,414 bp in length and contained major capsid genes, including penton base, hexon, and fiber. It was therefore used as a representative assembled sequence for read recruitment analysis (Figure 2A).

Median coverage breadth was 61.15% (range, 2.31%-100%) in pediatric BALF libraries and 0.796% (range, 0.596%-0.884%) in adult BALF libraries. Coverage breadth in the three environmental controls ranged from 0.788% to 1.194%. Among the 30 pediatric libraries, 20 covered at least 50% of the representative contig and seven covered at least 90% (Supplementary Table S2).

**Figure 2:**
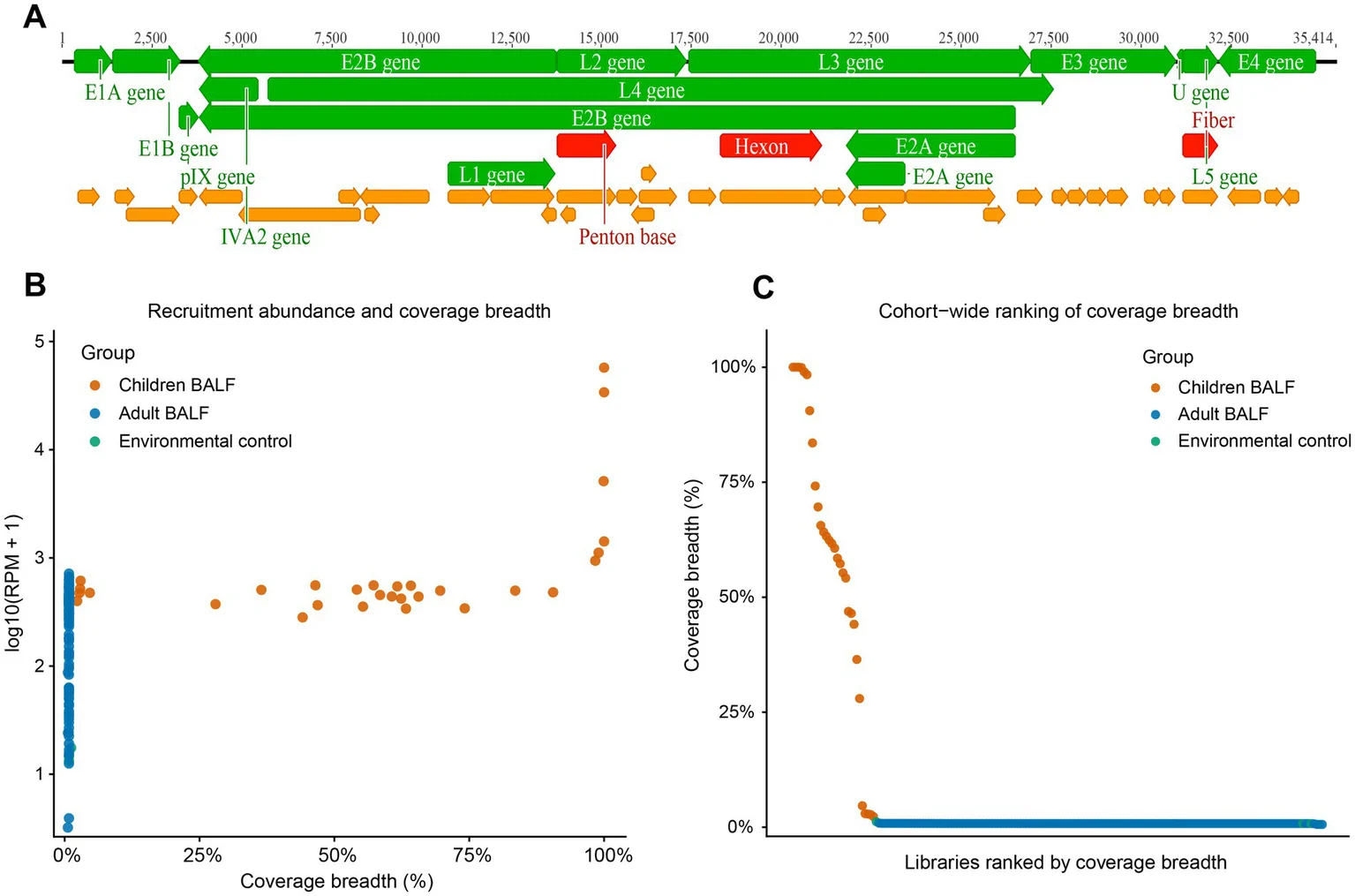
Genome organization and read recruitment features of the BALF-derived HAdV-B-related representative contig. **(A)**, Genome organization of the BALF-derived near-complete HAdV-B-related representative contig. Major capsid genes, including penton base, hexon, and fiber, are highlighted in red. Other genes are shown in green, and predicted ORFs are shown in orange. The horizontal scale indicates nucleotide positions along the 35,414-bp contig, and arrowheads indicate the orientation of annotated features. E and L denote early and late gene regions, respectively. Overlapping features are displayed on separate rows for clarity. **(B)**, Relationship between representative-contig RPM and coverage breadth after read recruitment. RPM represents the number of read pairs recruited to the representative contig per million input read pairs used for recruitment. Coverage breadth represents the percentage of bases in the representative contig covered by at least one read. Each point represents one library, and colors indicate sample groups. (**C)**, Cohort-wide distribution of coverage breadth after all libraries were ranked from high to low coverage breadth. Each point represents one library, and colors indicate sample groups. Coverage breadth was used to describe the distribution of reads across the representative contig and was not used as a clinical positivity threshold.

Read recruitment showed that reads from pediatric BALF libraries could be recruited to the representative contig, and reads from several pediatric samples covered broad regions of the contig. In the two-dimensional distribution of RPM and coverage breadth, some pediatric BALF libraries showed both higher representative-contig RPM and higher coverage breadth (Figure 2B). By contrast, adult BALF libraries showed a certain degree of read recruitment at the RPM level, but these reads generally covered a narrower region and were mainly located in the low-coverage-breadth range. Laboratory environmental controls also did not show broad contig coverage.

We next ranked all libraries according to coverage breadth. Libraries with higher coverage breadth were mainly from the pediatric BALF group, whereas most adult BALF libraries and environmental controls were concentrated in the low-coverage-breadth range (Figure 2C). These results indicated that representative-contig recruitment in pediatric BALF libraries showed broader contig-level read support. In this study, coverage breadth was used only to describe the distribution of reads across the representative contig and was not used as a clinical positivity threshold. Representative-contig recruitment should also not be interpreted as equivalent to clinical viral load or infection diagnosis.

To assess whether the low-coverage-breadth pattern was restricted to a particular adult clinical subgroup, representative-contig recruitment metrics were further summarized across the major adult diagnostic categories. Median coverage breadth remained approximately 0.8% across all adult categories, and no adult diagnostic subgroup showed a maximum coverage breadth exceeding 0.884% (Supplementary Table S1C). Thus, the low-breadth recruitment pattern was observed across the adult diagnostic categories summarized in Supplementary Table S1C.

### 3.3 Phylogenetic analysis of major capsid genes supported the placement of BALF-derived sequences within the HAdV-B-related lineage

To further determine the phylogenetic placement of BALF-derived HAdV-related sequences, we reconstructed phylogenetic trees based on three major capsid proteins: penton base, hexon, and fiber. Because some BALF-derived sequences were assembled fragments, phylogenetic analyses were performed using the shared overlapping amino acid regions between BALF-derived sequences and reference sequences.

The three protein trees placed the BALF-derived sequences within HAdV-B-related lineages (Figures 3A–C; Supplementary Table S3A). Among the 27 capsid protein sequences, 23 had best BLASTp matches to references annotated as HAdV-B3 and four to references annotated as HAdV-B7. The B3-related matches comprised eight penton base, seven hexon, and eight fiber sequences; the B7-related matches comprised three hexon sequences and one fiber sequence. The fiber reference QJX15980.1 is labeled Human mastadenovirus B in its record title but has serotype B3 recorded in its source metadata (Supplementary Table S3A). These are marker-level reference matches and do not constitute definitive whole-virus type assignments.

**Figure 3:**
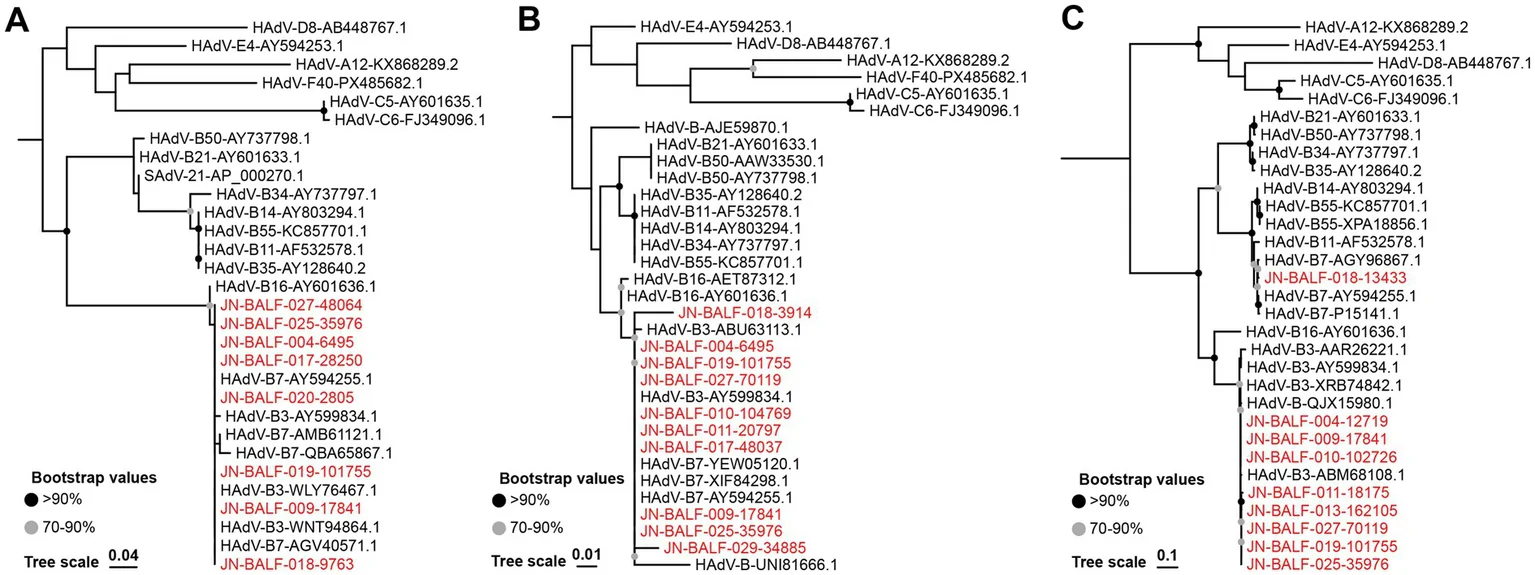
Phylogenetic analysis of BALF-derived HAdV major capsid protein sequences. **(A–C)**, Phylogenetic trees reconstructed based on penton base, hexon, and fiber protein sequences, respectively. BALF-derived HAdV-related sequences identified in this study are shown in red, and reference sequences are shown in black. Circles at internal nodes indicate bootstrap support values, and scale bars indicate amino acid substitutions per site. Phylogenetic analyses were performed using the shared overlapping amino acid regions between BALF-derived sequences and reference sequences.

Marker-level matches differed within three samples. JN-BALF-010 and JN-BALF-011 had B3-related fiber matches and B7-related hexon matches, whereas JN-BALF-018 had B7-related fiber and hexon matches and a B3-related penton base match. The corresponding markers were recovered from separate contigs. These results therefore do not establish that discordant markers belong to the same viral genome.

To place these genomic findings in clinical context, we summarized the available clinical information together with representative-contig recruitment metrics and capsid-marker best BLASTp matches for the 12 pediatric BALF samples represented in the phylogenetic analyses (Supplementary Table S3B). Eight of the 12 samples had adenovirus infection or adenoviral pneumonia recorded in the available clinical records, whereas four did not have such an annotation recorded. The clinical backgrounds were heterogeneous and frequently included other recorded respiratory pathogens or associated conditions. Given the limited sample size, incomplete or discordant marker-level information in some samples, and the lack of standardized clinical outcome data, no formal genotype–phenotype association analysis was performed. Overall, MEGAN read classification, representative-contig read recruitment, and phylogenetic analysis of major capsid genes jointly supported HAdV-B-related mNGS signals in clinically selected pediatric BALF samples.

## 4 Discussion

In this study, we characterized *Adenoviridae*-related signals in BALF mNGS data from pediatric and adult clinical cohorts. We observed higher *Adenoviridae* read-classification abundance in several pediatric BALF libraries and then examined these signals using HAdV-related contigs, representative-contig read recruitment, and phylogenetic analyses of penton base, hexon, and fiber. The results support HAdV-B-related sequence signals in a subset of clinically selected pediatric BALF samples, with support from assembly, broad read coverage in several libraries, and phylogenetic placement.

HAdV is one of the important viral pathogens associated with respiratory infections and pneumonia in children. Previous studies have shown that HAdV infection can lead to a wide range of clinical manifestations, from mild upper respiratory tract infection to severe pneumonia, and pediatric patients remain a major population of interest in HAdV pneumonia research (33, 34). In pediatric pneumonia or lower respiratory tract infection cohorts in China, HAdV-B3 and HAdV-B7 have frequently been reported as common types (35, 36). Some studies have also shown that HAdV-B7-related infection may present with more severe clinical features than HAdV-B3-related infection (37). The HAdV-B-related mNGS signals identified in pediatric pneumonia BALF in the present study are consistent with previous reports on the epidemiological features of pediatric respiratory HAdV-B. Because the samples in this study were derived from BALF, our findings also suggest that HAdV-B-related signals in lower respiratory tract samples deserve attention in virome studies of pediatric pneumonia.

This study further incorporated assembly and read recruitment results to strengthen signal interpretation. mNGS read classification can provide rapid and broad information for pathogen screening, but low-abundance reads, conserved protein regions, and database similarity can all affect taxonomic interpretation (38, 39). In this context, assembled HAdV-related contigs provide longer sequence backgrounds than individual reads. Representative contig read recruitment can further assess whether the assembled sequence is supported by the original sequencing data from the perspective of read distribution. In this study, reads from some pediatric BALF libraries were not only recruited to the HAdV-B-related representative contig, but also covered broad regions of the contig. This finding indicates that HAdV-B-related signals in pediatric BALF had clearer contig-level read support.

Coverage breadth was an important interpretive metric in this study. RPM reflects the abundance of recruited reads, but it cannot by itself distinguish whether reads are distributed across the contig or concentrated in a local conserved region. We observed a certain degree of representative-contig RPM signal in adult BALF libraries, but these signals were mainly located in the low-coverage-breadth range. By contrast, several pediatric BALF libraries showed both higher RPM and higher coverage breadth. This difference suggests that HAdV-B-related signals in pediatric samples were more consistent with broad contig support, whereas local recruitment in adult samples was more appropriately interpreted as a low-coverage-breadth mapping signal. This approach reduces the risk of overinterpretation based solely on the number of mapped reads and provides a description that is more suitable for virome data.

The pediatric and adult cohorts should not be regarded as clinically matched populations. In addition to age, the two cohorts differed in clinical composition and sampling center. The pediatric cohort was predominantly composed of patients with pneumonia or bronchopneumonia and frequently included recorded respiratory co-pathogens, whereas the adult cohort represented a broader range of clinical conditions. Accordingly, the more prominent HAdV-related signals observed in the pediatric cohort cannot be attributed to age alone and may also reflect differences in underlying disease, co-infection background, clinical indications for BALF sampling, and source population. Nevertheless, representative-contig coverage breadth remained consistently low across the major adult diagnostic categories, and the low-breadth pattern was observed across the adult diagnostic categories summarized in Supplementary Table S1C. We therefore interpret these findings as cohort-level differences in metagenomic signal patterns rather than as estimates of age-specific HAdV prevalence or an independent effect of age.

Phylogenetic analysis provided additional lineage information. Molecular typing of HAdV should not rely on a single short fragment. Hexon, penton base, and fiber are major capsid genes commonly used for HAdV typing, and combined analysis of these genes can improve type-related interpretation (40). Sequence variation in the hexon neutralization determinants and fiber knob has also been used for molecular classification of HAdV prototypes (41). In this study, BALF-derived sequences from all three capsid proteins were placed within HAdV-B-related lineages. Best-hit reference annotations were predominantly HAdV-B3, with fewer HAdV-B7 matches, but marker-level matches differed in several samples.

HAdV can undergo genetic recombination, and different genomic regions may show discordant evolutionary relationships (42, 43). Recombination, mixed infection, assembly artifacts, and limited discriminatory information in partial protein regions are possible explanations for discordant marker-level matches, but these possibilities cannot be distinguished with the present data. Major capsid-gene sequences can support identification of known HAdV types, whereas whole-genome data are particularly important for characterizing novel types and complex recombinants (16, 40). In this dataset, incomplete marker coverage, discordant reference matches, and the lack of genome-level linkage among markers limited definitive type assignment. We therefore retained HAdV-B-related as the overall lineage description.

The available clinical and genomic data were insufficient to assess whether capsid-marker patterns were associated with clinical presentation. Previous studies have reported type-specific differences in pediatric respiratory HAdV infection, including more severe disease associated with HAdV-7 than HAdV-3 in some cohorts (37). However, only a subset of pediatric BALF samples yielded capsid sequences suitable for phylogenetic analysis, several patients had recorded co-pathogens or other associated conditions, and definitive type assignments and standardized clinical outcome data were not available. We therefore present the clinical and genomic information descriptively and do not draw conclusions regarding type-specific disease severity.

BALF provided a valuable sampling window for this study. Compared with nasopharyngeal or throat swabs, BALF is closer to the pulmonary lesion site and can provide more direct lower respiratory tract information for pneumonia pathogen analysis. Previous studies have shown that BALF mNGS can improve pathogen spectrum identification in patients with pneumonia and has clinical value in pediatric pneumonia and severe pneumonia (44, 45). The present study was not a clinical diagnostic trial, but a virome analysis based on residual BALF samples. This design allowed us to re-examine the distribution, assembly support, and phylogenetic placement of HAdV-related signals in lower respiratory tract samples. Compared with detecting viral nucleic acids only in nasopharyngeal samples, BALF mNGS can provide more direct sequence evidence for viral signals in the lower respiratory tract of patients with pneumonia.

This study also included laboratory environmental surface controls. In virome studies, low-biomass samples, reagent background, and laboratory environmental nucleic acids can affect interpretation of low-abundance viral signals (46, 47). The environmental controls were therefore used as limited background references rather than as proof of the absence of contamination. They did not show coverage breadth comparable to that of the broadly covered pediatric samples. This provides limited reassurance against the background represented by these controls, but does not exclude reagent contamination or cross-sample carryover.

For clinically selected BALF samples, assembly and coverage breadth can help assess the support for viral read assignments rather than relying on read-level classification alone. Residual specimens may also provide sequence data from clinically selected pediatric patients and help identify candidate samples for targeted typing or hospital-based genomic surveillance. These data do not estimate population prevalence or establish transmission links. Prospective studies should combine quantitative assays, genome sequencing, and standardized clinical outcomes to clarify the clinical and epidemiological significance of HAdV-B-related signals.

### 4.1 Limitations

This study also has several limitations. First, this study analyzed residual BALF specimens and available clinical information from clinically selected patients. The pediatric and adult cohorts differed in sample size, clinical composition, and sampling center and were not designed as clinically matched populations. Therefore, the observed between-cohort differences cannot be attributed to age alone, and the results should be interpreted primarily as differences in metagenomic signal patterns rather than as estimates of age-specific HAdV prevalence. Second, this study did not include further qPCR validation. The main reason was that the remaining BALF volume was insufficient after viral particle enrichment, nucleic acid extraction, and library preparation, which prevented additional targeted qPCR or viral load testing. Third, the phylogenetic analyses were based on partially overlapping regions of major capsid proteins rather than complete HAdV genomes from each sample. This strategy can support HAdV-B-related lineage placement, but it is not sufficient for strict type assignment or recombination analysis. Fourth, this study did not include viral culture, serology, longitudinal samples, or complete clinical outcome data. We therefore could not further evaluate viral replication activity, disease-course dynamics, or the direct relationship between HAdV-B-related signals and pneumonia severity. Fifth, read recruitment was performed using a single BALF-derived HAdV-B-related representative contig. This strategy allowed a consistent sample-level comparison of recruitment abundance and coverage breadth, but it may preferentially capture sequences closely related to the representative contig and cannot fully resolve the diversity of all HAdV-B-related sequences in the cohort. Coverage breadth also depends on sequencing depth and sequence similarity, and RPM does not provide an absolute measure of viral load. In addition, the environmental controls used in this study represented laboratory surface controls and did not replace extraction blank or library blank controls.

Future studies could reserve sufficient BALF or nucleic acid material in prospective designs for qPCR, ddPCR, or targeted amplicon sequencing validation. If viral nucleic acid quantity is sufficient, future work could also perform whole-genome reconstruction and recombination analysis to further resolve the precise type and evolutionary features of BALF-derived HAdV-B-related sequences. For clinical correlation, future studies should integrate disease timing, imaging findings, immune status, co-infections, treatment information, and clinical outcomes to further evaluate the significance of HAdV-B-related mNGS signals in pediatric pneumonia.

## 5 Conclusion

This analysis identified stronger *Adenoviridae* read-classification signals in pediatric than in adult clinical BALF libraries. Several pediatric samples showed broad coverage of a BALF-derived HAdV-B-related representative contig, and capsid protein phylogenies supported HAdV-B-related lineage placement. These findings provide genomic information from clinically selected lower respiratory tract samples. They do not establish an independent age effect, population prevalence, or type-specific disease severity.

## Data Availability

The raw sequencing data generated in this study are available under BioProject accession PRJCA061470, and the assembled contigs have been deposited in GenBase under accession numbers C_AA474481.1–C_AA474499.1. Both datasets are hosted by the National Genomics Data Center, China National Center for Bioinformation.
